# Maternal Overweight and Obesity and Their Effect on the Growth of the Newborn During the First Six Months of Life

**DOI:** 10.7759/cureus.64867

**Published:** 2024-07-18

**Authors:** Jorge I Zurutuza, Mario Caba, Jaime Morales-Romero, Mario D Caba-Flores, Rubi Viveros-Contreras

**Affiliations:** 1 Epidemiology and Biostatistics, Centro de Investigaciones Biomédicas, Universidad Veracruzana, Xalapa, MEX; 2 Neurosciences, Centro de Investigaciones Biomédicas, Universidad Veracruzana, Xalapa, MEX; 3 Instituto de Salud Pública, Universidad Veracruzana, Xalapa, MEX; 4 Facultad de Medicina, Universidad Autónoma de Nuevo León, Monterrey, MEX; 5 Nutrition, Centro de Investigaciones Biomédicas, Universidad Veracruzana, Xalapa, MEX

**Keywords:** perinatal outcomes, macrosomia, cesarean section, obesity, gestational weight gain, pre-pregnancy body mass index, maternal obesity, metabolic processes, breast milk expression, exclusive breast feeding

## Abstract

Introduction: Maternal overweight and obesity during pregnancy have been shown to have multiple negative effects on the mother's health, which can even affect the infant's growth by increasing weight gain and altering various indicators, such as weight for age, length for age and weight for length. While breast milk on the other hand reduces these risks, and it’s the best and most complete food for the newborn. It’s a dynamic fluid capable of being modified to meet the needs of each stage of the newborn, but despite this capacity and the fact that maternal body mass index can have an impact on its components, through complex biological mechanisms, it manages to reduce the negative effects accumulated during pregnancy and even promotes a healthy state in the baby. In a country like Mexico, where overweight and obesity affect a large part of the population, it is important to study their causes and which could be the effect of this increased maternal overweight during pregnancy and lactation on newborns.

Objective: Identify the alterations associated with increased maternal body mass index during pregnancy and breastfeeding on mothers' health and their possible effect on the growth of the newborn during the first six months of life.

Material and methods: This was a prospective cohort study. Forty-two healthy binomials (mother and child), without problems during delivery and without serious illnesses during the breastfeeding period, were included. Maternal body mass index at the beginning of pregnancy allowed us to create two comparison groups between mothers: one with adequate weight, another with overweight or obesity. Follow-up was carried out once a month during the first six months of life, evaluating the somatometric development of mothers and children. All mothers completed the six-month period of exclusive breastfeeding.

Results: There were differences between both groups of women. The one that included overweight and obese women compared to the group of women with adequate weight had a higher number of pregnancies, abortions, plasma glucose levels in the third trimester of pregnancy, and a lower number of prenatal control visits and plasma platelet levels (all with p<0.05). Regarding the baby's growth, there was a difference between the weight for length classification at 60-, 120-, 150- and 180-day follow-ups. The group to which the mother was assigned with respect to her body mass index at the beginning of pregnancy (adequate weight group and overweight/obese group) was the only factor associated with the risk of the baby being overweight according to weight for length indicator at the 180-day follow-up, with an OR = 5.2 (95%CI 1.02-26.59).

Conclusions: Maternal overweight and obesity during pregnancy have a negative effect on the mother's health and baby's weight gain in its weight-for-length classification during the first six months of life. Although breastfeeding has been shown to have a positive effect on the growth of the baby, exposure to a higher maternal body mass index during pregnancy triggers important metabolic alterations that promote the development of diseases. It is important to establish weight control guidelines in women who wish to become pregnant to reduce the negative effects on the mother and offspring.

## Introduction

Overweight and obesity, which implies a body mass index (BMI) >25 Kg/m^2^, are a major public health problem in Mexico. Their prevalence in the general population over 20 years of age is 75.2% [1]. In children the outlook seems favorable, since for those under two years of age the prevalence of overweight and obesity is lower, being 5.7% (95%CI 4.1%-8%), however, this increases with age, being in the group from two to five years of 8.7% (95%CI 6.2%-12.2%), which eventually, as they grow, leads to the prevalence for adults, where three out of four suffer from some of these weight alterations [1,2]. Likewise, it should not be forgotten that Mexico also has malnutrition, reducing the margin of those who are at an adequate weight.

One of the simplest, fastest and cheapest ways to evaluate and classify maternal weight is the BMI [3]. The problem appears in the difficulty of monitoring newborns, since it may be inappropriate to base only on weight to evaluate growth. To unify the appropriate criteria for monitoring, the World Health Organization (WHO) created indicators to be able to carry out more accurate monitoring of babies, using age, weight and length (from birth to two years, then height will be used) to classify growth [4].

During pregnancy, overweight and obesity are considered altered metabolic states, which have an intra-utero effect on the fetus. The mechanisms that trigger the alterations in the baby have not been clearly identified, but it is considered that alterations in maternal hormones (such as insulin, glucagon, ghrelin, leptin, etc), the inflammatory status and alterations in micro and macronutrients could be the cause [5].

The described effects on the mother and baby of high maternal BMI during pregnancy are greater baby weight for gestational age, greater probability of failure to progress in labour, difficulty in initiating breastfeeding due to multiple causes, maternal weight gain difficult to lose and higher number of pregnancy losses [6,7]. It is important to mention that overweight and obesity during pregnancy have been associated with greater weight in the infant, which can even lead to obesity during adult life [7].

On the other hand, human breast milk is a fluid with a high capacity for adaptation during the lactation period. Colostrum, transitional milk and mature milk are different in contents and characteristics [8], it is also modified according to the needs of the newborn [9] and with maternal characteristics [10]. These modifications benefit maternal health and infant growth in the short and long term [11,12]. These changes are necessary and are identified through the breastfeeding process itself and generate important changes in the milk content [10,13]. These benefits are so important that the WHO recommends exclusive breastfeeding for the first six months of life and complementary breastfeeding for at least two years [14].

The effect of breast milk on the development of the newborn is mediated by the great diversity of components it contains (macronutrients and bioactive compounds), which are used in biological processes and regulate metabolic pathways [15]. Despite this great susceptibility to change, it only works to increase the beneficial effect on the baby [16], and although changes in its composition have been reported in maternal overweight and obesity [17-19], it continues to have a positive effect on the baby's health, significantly reducing the risk of overweight during lactation and in childhood [16,19-22].

Once the need to study the effect of overweight and obesity during pregnancy and its metabolic alterations was identified, due to its high prevalence in Mexico, we seek to identify whether breastfeeding can reduce the negative effects. Therefore, the objective of this project is to identify the alterations associated with increased maternal BMI during pregnancy and breastfeeding on mothers’ health and their possible effect on the growth of the newborn during the first six months of life.

## Materials and methods

Study design

A prospective cohort study was carried out in which 42 healthy binomials of mothers and newborns from childbirth up to six months were included. The study population belonged to a second-level hospital and was invited to participate during the third trimester of pregnancy prior to delivery care, under informed consent. The grouping method was according to the BMI at the beginning of pregnancy, which was recorded in the mothers' prenatal control follow-up records. Two groups were formed, one with mothers with adequate weight (BMI >19 and <25 Kg/m^2^) and the second with overweight or obese mothers (BMI > 25 Kg/m^2^). To ensure that the mothers continued in the assigned group during the postpartum and breastfeeding, measurements (weight and height) were taken during the follow-up time and the difference between the BMI means was tested by statistical tests. The inclusion criteria were a mother without metabolic (gestational diabetes and hypothyroidism) or blood pressure alterations during pregnancy, childbirth and the postpartum period (except for overweight or obesity) and a child without congenital malformations, digestive or metabolic alterations, or allergy to breast milk and without alterations in the neonatal screen. Once entered into the study, sociodemographic data, as well as somatometric data of the newborn at the time of delivery were collected. 

Follow-ups were carried out once a month for six months. The data obtained at birth and at each follow-up were weight, length and head circumference of the child and maternal weight, height, hip and waist were also measured. The Graffar-Méndez-Castellanos scale of Socioeconomic Level (GMC-SE) was applied to classify the mothers [23]. To classify growth and development, the growth indicators of the WHO were used, which use weight for length, length for age, weight for age and head circumference diameter. The exit criterion from the cohort was the suspension of exclusive breastfeeding, maternal or infant death. There were no losses in the cohort since mothers at risk of abandonment were trained in breastfeeding issues according to the areas of opportunity identified. The protocol was approved by the committees of Xalapa Regional Hospital Dr. Luis F. Nachón where the research work was carried out with approval number 10/21. The project was carried out in the population of influence of the Xalapa Regional Hospital Dr. Luis F. Nachón by personnel from the Biomedical Research Center of the Universidad Veracruzana. This work was supported by grant No. 783848 assigned to JIZL, by the Consejo Nacional de Humanidades, Ciencias y Tecnologías (CONAHCYT).

Statistics analysis

Statistical analysis was carried out for categorical variables through Pearson’s X2, X2 with Mantel-Haenzel correction and Fisher's exact test, according to the assumptions. While for continuous variables, the Student's t-test or U-Mann Whitney test was used, in accordance with the assumption of normality and homoscedasticity. In addition, a correlation analysis was performed using the Spearman correlation test (S) for the baby's weight during the follow-ups and the rest of the mother and baby variables. Finally, a binary logistic regression model was carried out in which the baby's classification of weight for length at the 180-day follow-up was used as a dependent variable, with one group being those with “adequate weight” and another being patients “at risk of overweight", while the independent variables were the classification group of mothers with adequate weight and mothers with overweight and obesity at beginning of pregnancy, maternal plasma glucose (identified in the third trimester of pregnancy), maternal age on mg/dL and the weight of the newborn at birth. Statistical analysis was performed using SPSS Statistics version 29 software (IBM Corp., Armonk, NY, USA).

## Results

There were no losses to follow-up. Regarding the general characteristics of the mothers, the mean age was 23.6 (standard deviation (SD) = 5.2) years, 83.4% (35 cases) had a partner (stable, with or without civil union), 71.4% (30 cases) have high school or less, 88.1% (37 cases) are housewives, in 38.1% (16 cases) the daily family income is less than a Mexican minimum daily wage (15 dollars or less), 57.1% (24 cases) had overcrowded homes, with four or more people per room.

None of the mothers had disabilities or chronic-degenerative diseases, none of them smoked, and only one woman (2.4%) consumed alcohol frequently. Regarding the social conditions of the mothers, 21.4% (nine cases) have a tin or wooden roof, 2.4% (one case) has wooden walls and an earthen floor, 11.9% (five cases) do not have running drinking water, so it is obtained directly from wells or rivers, 23.8% (10 cases) do not have a direct connection to the drainage network, so they use septic tanks, and 4.8% (two cases) eliminate their garbage through the incineration and not in the government collection network. In the gynecological-obstetric history, 40.5% (17 cases) did not consider the pregnancy planned, in 50% (21 cases) it was their first pregnancy, 81% (34 cases) had at least five prenatal control visits, with the average number of consultations being seven (SD = 2.5). The GMC-SE instrument identified that 66.7% (28 cases) are in the fourth quintile (the penultimate lowest).

About the children, 52.4% are female babies (22 cases), the mean of gestational age was 39.16 (SD = 1.1) weeks, the mode of delivery mainly was abdominal via cesarean section, with 69% (29 cases), the most frequent cause of the surgical event was the lack of progression of labor (dynamic dystocia) with 22 of the 29 cases (75.86%). Concerning the newborns anthropometric measures at birth, the averages were: weight of 3208.8 (SD =449.19) grams, height of 49.45 (SD = 2.62) centimeters, and head circumference of 34.94 (SD = 1.29) centimeters. Skin-to-skin contact at birth occurred in 57.1% (24 cases), and breastfeeding initiation in the first hour occurred in 52.4% (22 cases).

In the analysis according to the classification of maternal weight. The difference between both groups was verified with respect to the BMI of the mothers at the beginning of pregnancy and every follow-up. It is, throughout the entire follow-up the difference between groups continued, confirming that the difference existed throughout the pregnancy and the period of exclusive breastfeeding. Then, the baby was exposed to maternal overweight and obesity from pregnancy and up to six months after birth. The maternal sociodemographic and housing variables, as well as those regarding pregnancy, childbirth and postpartum care, did not have significant differences (Table 1).

**Table 1:** Distribution of the maternal sociodemographic and housing characteristics, prenatal care, childbirth and puerperium, according to maternal BMI at the beginning of pregnancy Statistical tests used: ^a^Pearson’s X^2^, ^b^X^2^ with Mantel-Haenszel correction, ^c^Fisher’s Exact Test (1-sided). Definitions: AW: Adequate weight = BMI >19 y < 25 Kg/m^2^, SO: Overweight and obesity = BMI > 25 Kg/m^2^, BMI: Body Mass Index, GMC-SE: Graffar-Méndez-Castellanos scale of Socioeconomic Level *p value <0.05

Variable		Category			Adequate weight (PA) (n = 21)			Overweight and obesity (SO) (n = 21)			p value
					n		%	n	%		
Daily income (15 dollars a day)		Less than one salary per day			1		4.8	3	14.3		0.65^b^
		Equal to one salary per day			9		42.9	3	14.3		
		Greater than one salary per day			11		52.3	15	71.4		
Civil status		Single			2		9.5	5	23.8		0.21^c^
		Married (with or without civil union)			19		90.5	16	76.2		
Mother’s education		Primary School			3		14.3	5	23.8		0.99^b^
		Secondary School			11		52.4	7	33.3		
		High School or higher			7		33.3	9	42.9		
Mother’s occupation		Housewife			18		85.7	19	90.5		0.5^c^
		Remunerated job			3		14.3	2	9.5		
Construction material: roof		Durable material (concrete)			18		85.7	15	71.4		0.23^c^
		Non-durable material (aluminum sheet, wood)			3		14.3	6	28.6		
Construction material: walls		Durable and resistant material (brick)			6		28.6	11	52.4		0.12^a^
		Durable but not resistant material (wood and others)			15		71.4	10	47.6		
Construction material: floor		Durable material (concrete)			21		100.00	20	95.2		0.5^c^
		Non-durable material (ground, wood)			0		0.00	1	4.8		
Drinking water fountain		Tubing water			19		90.5	18	85.2		0.5^c^
		Water from non-potable sources			2		9.5	3	14.8		
Drainage type		Sewer system			17		80.9	15	71.4		0.36^c^
		Septic tank or latrine			4		19.1	6	28.6		
Garbage collection type		Public garbage collection network			20		95.2	20	95.2		0.76^c^
		Burning and burying garbage			1		4.8	1	4.8		
Newborn sex		Male			8		38.1	12	57.1		0.22^a^
		Female			13		61.9	9	42.9		
Mode of delivery		Eutocic delivery			8		38.1	5	23.8		0.32^a^
		Caesarean section			13		61.9	16	76.2		
Cause of cesarean section		Lack of progression of labor (dynamic dystocia)			11		52.4	11	52.4		0.29^c^
		Other			2		9.6	5	23.8		
Skin-to-skin contact at birth		No			7		33.3	11	52.4		0.35^c^
		Yes			14		66.7	10	47.6		
Initiation of breastfeeding in first hour of life		No			8		38.1	12	57.1		0.35^c^
		Yes			13		61.9	9	42.9		
Milk intake at night		No			1		4.7	2	9.5		0.5^c^
		Yes			20		95.3	19	90.5		
Planned pregnancy		No			7		33.3	10	47.6		0.35^a^
		Yes			14		66.7	11	52.4		
Illness during pregnancy		No			6		28.6	9	42.9		0.34^a^
		Yes			15		71.4	12	57.1		
Socioeconomic level based on GMC-SE instrument score		Upper middle stratum			1		4.8	0	0.00		0.39^b^
		Lower middle stratum			7		33.3	6	28.6		
		Working class			13		61.9	15	71.4		

Regarding the differences between maternal weight groups, we found a statistically significant difference were the difference between the number of prenatal consultations, with the number of consultations which was higher in the group of women with adequate weight. Likewise, in the gynecological-obstetric characteristics, the women with obesity had a greater number of births and abortions. Furthermore, it can be seen in Table 2that women in the overweight and obese group had a significantly higher plasma level of glucose and a low platelet count (p <0.05 in all cases).

**Table 2:** Distribution of the maternal somatometric characteristics and newborn developmental characteristics at birth and during follow-up periods, according to maternal BMI at the beginning of pregnancy Statistical tests used: ^a^Student’s t test for independent groups, ^b^U-Mann-Whitney test. Definitions: AW: Adequate weight = BMI >19 y < 25 Kg/m2, SO: Overweight and obesity = BMI > 25 Kg/m2, BMI: Body Mass Index, GMC-SE: Graffar-Méndez-Castellanos scale of Socioeconomic Level *p value <0.05

Variable	Maternal group	Mean	Standard deviation	Median	Interquartile range	P value
Maternal BMI at the beginning of pregnancy	AW	21.94	1.31	21	7	0.01^b^*
	SO	20.21	6.133	23	6	
Maternal BMI at day 180 of follow-up	AW	22.45	1.39	22.55	2.09	0.01^b^*
	SO	29.66	5.66	28.19	4.99	
Maternal Age	AW	23.43	5.96	22.07	2.49	0.43^b^
	SO	23.81	4.48	27.56	5.54	
Gestation weeks	AW	39.3	0.85	39.1	1	0.2^b^
	SO	39	1.32	39.2	2	
Number of prenatal check-ups	AW	7.52	2.86	8	3	0.048^a^*
	SO	6.24	1.92	6	4	
Pregnancy number (total)	AW	1.33	0.48	1	1	0.01^b^*
	SO	1.68	1.12	2	2	
Number of eutocic deliveries (total)	AW	0.57	0.68	0	1	0.73^b^
	SO	0.62	0.97	0	1	
Number of cesarean sections (total)	AW	0.76	0.7	1	1	0.07^b^
	SO	0.83	0.89	1	2	
Number of previous abortions (total)	AW	0	0	0	0	0.02^b^*
	SO	0.33	0.66	0	1	
Glucose levels in the third trimester of pregnancy (mg/dL)	AW	79.86	7.9	79	10	0.01^a^*
	SO	86.29	8.64	86	12	
Creatinine levels in the third trimester of pregnancy (mg/dL)	AW	0.59	0.12	0.6	0.14	0.49^a^
	SO	0.59	0.12	0.57	0.21	
Hemoglobin levels in the third trimester of pregnancy (mg/dL)	AW	11.73	1.04	12	1.6	0.33^a^
	SO	11.87	1.04	12.2	1.2	
Platelets levels in the third trimester of pregnancy (count/mL)	AW	244857.14	46011.18	250000	58500	0.03^a^*
	SO	220142.86	36701.88	223000	656500	
Leukocytes levels in the third trimester of pregnancy (count/mL)	AW	7627.81	1999.29	7900	2110	0.9^b^
	SO	7836.76	2449.23	7850	3620	
GMC-SE instrument score	AW	12.76	1.87	14	3	0.31^b^
	SO	13.43	1.75	14	4	

**Figure 1:**
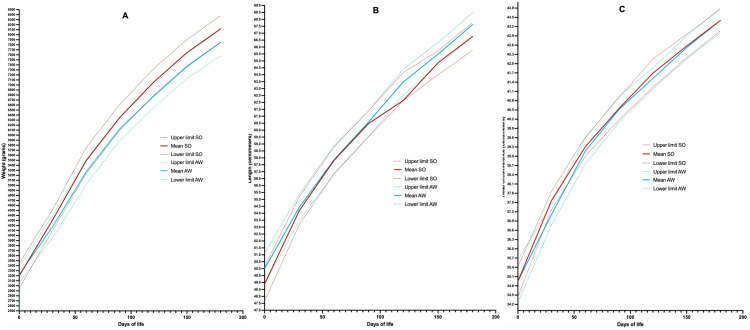
Monthly monitoring of the development of the newborn A) Weight (grams), B) Length (centimeters) and C) Head circumference (centimeters). The mean and 95%CI for this were plotted. In red, children of mothers from the overweight-obesity group, in blue, of mothers with adequate weight. According to weight at the beginning of pregnancy. Definitions: AW: Adequate weight = BMI >19 y < 25 Kg/m^2^, SO: Overweight and obesity = BMI > 25 Kg/m^2^

In relation to the characteristics section of the newborn (Table 2)*, *the head circumference in centimeters 30 days after birth was greater in the overweight and obese group. In relation to the weight in grams, length in centimeters and the rest of the head circumference measurements in centimeters there was no difference between groups (Figure 1). However, there was a trend in the follow-up at 120 days, 150 days (both with p = 0.05) and 180 days (with p = 0.07). In Figure 1 it is also possible to observe a greater weight of the children of women in the overweight and obesity group (no statistical difference), while the children of mothers in the adequate weight group tend more to have a lower weight and reduced confidence intervals.

Since no differences were found in the weight of newborns in grams, the WHO classification system considers a more detailed analysis of weight and their relationship to other measures to evaluate growth. This classification evaluates weight for age, length for age and weight for length. As shown in Table 3 we found that at 150-day follow-up, babies from maternal adequate weight group have a greater probability of having a greater length for age, while the maternal overweight and obesity group has a greater probability of having a lower length for age (p < 0.01). Also weight for length classified as overweight risk were more prevalent in the maternal overweight and obese group (all with p < 0.05) at 60-, 120-, 150- and 180-day follow-ups in comparison with the adequate weight group. Finally, at 180-day follow-up, there was a greater probability of risk of overweight according to weight for length (p = 0.03) in the maternal overweight and obesity group in comparison with the adequate weight group.

**Table 3:** Differences between groups of cases categorized by weight for age, length for age and weight for length by follow-up period Statistical tests used: X2 with Mantel-Haenszel correction The classification of weight for age, length for age and weight for length was carried out based on WHO graphs and tables. Definitions: AW: Adequate weight = BMI >19 y < 25 Kg/m^2^, SO: Overweight and obesity = BMI > 25 Kg/m^2^, BMI: Body Mass Index. *p-value < 0.05

Variable	Category	30-day follow-up				60-day follow-up				90-day follow-up				120-day follow-up				150-day follow-up				180-day follow-up			
		AW		SO		AW		SO		AW		SO		AW		SO		AW		SO		AW		SO	
		n	%	n	%	n	%	n	%	n	%	n	%	n	%	n	%	n	%	n	%	n	%	n	%
Weight for age	Under weight	2	9.5	1	4.8	2	9.5	0	0.0	0	0	0	0	0	0	0	0	0	0	0	0	0	0	0	0
	Proper weight	18	85.7	19	90.5	18	85.7	18	85.7	20	95.2	18	85.7	20	95.2	18	85.7	20	95.2	17	81.0	20	95.2	17	81.0
	Risk of overweight	1	4.8	1	4.8	1	4.8	3	14.3	1	4.8	3	14.3	1	4.8	3	14.3	1	4.8	4	19.0	1	4.8	4	19.0
	p-value	0.66				0.1				0.3				0.3				0.16				0.16			
Length for age	Low length	2	9.5	2	9.5	0	0.0	3	14.3	1	4.8	3	14.3	0	0.0	3	14.3	0	0.0	5	23.8	0	0.0	4	19.0
	Appropriate length	18	85.7	18	85.7	18	85.7	17	81.0	17	81.0	17	81.0	17	81.0	16	76.2	17	81.0	16	76.2	17	81.0	15	71.4
	Longest length	1	4.8	1	4.8	3	14.3	1	4.8	3	14.3	1	4.8	4	19.0	2	9.5	4	19.0	0	0.0	4	19.0	2	9.5
	p-value	0.99				0.06				0.16				0.1				<0.01*				0.06			
Weight for length	Under weight	7	33.3	3	14.3	4	19.0	1	4.8	1	4.8	0	0.0	4	19.0	0	0.0	0		0	0	1	4.8	0	0.0
	Proper weight	13	61.9	16	76.2	16	76.2	15	71.4	18	85.7	15	71.4	16	76.2	17	81.0	20	95.2	13	61.9	17	81.0	12	57.1
	Risk of overweight	1	4.8	2	9.5	1	4.8	5	23.8	2	9.5	6	28.6	1	4.8	4	19.0	1	4.8	8	38.1	3	14.3	9	42.9
	p-value	0.15				0.04*				0.08				0.02*				<0.01*				0.03*			

Regarding the correlations, maternal BMI at the beginning of pregnancy correlates with the number of pregnancies (S = 0.47, p<0.01), the number of cesarean sections (S = 0.31, p = 0.047), and the numbers of glucose in the third trimester of pregnancy (S = 0.34, p = 0.03). 

Something important to mention is that if the mother begins pregnancy with a higher BMI, at 180 days postpartum she will continue with a higher BMI and weight. This is observed in the correlations between BMI at the beginning of pregnancy and BMI at 180-day follow-up (S = 0.92, p < 0.01), and maternal weight at 180-day follow-up (S = 0.81, p < 0.01).

On the other hand, the weight of the newborn in grams at birth is correlated with gestational age (S = 0.33, p = 0.03), number of maternal pregnancies (S = 0.35, p = 0.02), number of maternal cesarean sections (S = 0.38, p = 0.01), number of maternal abortions (S = 0.4, p < 0.01), maternal creatinine in the third trimester of pregnancy (S = 0.31, p = 0.04) and the score on the GMC-SE instrument (S = 0.32, p = 0.04)

Something similar happens to mothers in babies: if they have a higher weight in grams at birth, this higher weight than the average will continue until 180 days of follow-up. A correlation exists in the weight between these two moments in the baby's life (S = 0.48, p < 0.01).

Finally, the baby's weight at 180 days was correlated with: the baby's weight during the previous follow-ups (at birth -S = 0.37, p < 0.01-, 30 days -S = 0.49, p < 0.01-, 60 days -S = 0.67, p < 0.01-, 90 days -S = 0.74, p < 0.01- and 150 days -S = 0.82, p < 0.01-), as well as with the baby's head circumference in all the follow-ups (at 30 days -S = 0.42, p < 0.01-, 90 days -S = 0.36, p = 0.02-, 120 days -S = 0.39, p = 0.01-, 150 days -S = 0.4, p < 0.01- and 180 days -S = 0.4, p < 0.01-).

Finally, the binary logistic regression model (Table 4), indicates that the maternal group according to BMI (adequate weight group or overweight and obese group) is the highest risk factor for infant risk of overweight at 180 days of follow-up (OR = 5.203, 95%CI 1.018-26.59).

**Table 4:** Binary logistic regression model: Factors for the risk of overweight in offspring Nagelkerke's R^2^ 0.15, and Cox & Snell's R^2^ 0.10. Dependent variable: classification of weight at 180 days follow-up based on WHO graphs and tables. Definitions: AW: Adequate weight = BMI >19 y < 25 Kg/m^2^, SO: Overweight and obesity = BMI > 25 Kg/m^2^. BMI: Body Mass Index. *p value < 0.05

Variable	B	p value	Odds ratio (OR)	95%CI OR	
				Lower	Upper
Maternal group (AW or OS)	1.65	0.048*	5.203	1.018	26.59
Glucose levels in the third trimester of pregnancy (mg/dL)	-0.021	0.64	0.979	0.894	1.072
Newborn weight at birth (grams)	0.001	0.98	1	0.99	1.002

## Discussion

The present work implies that there is a relationship between maternal overweight and obesity during pregnancy and greater weight for length in the newborn. Despite the benefit of breastfeeding in controlling the metabolic alterations that result in exposure of the fetus to obesity during pregnancy, they may not be sufficient to reduce the risk.

The causes of a higher BMI in the women in the study may be due to the difficulty of losing the weight gained with each pregnancy, so this would explain a greater number of pregnancies in the group of overweight and obese women compared to the group of women with adequate weight. The intrauterine effect of overweight and obesity leads to an increase in the risk of overweight and obesity in the baby, in accordance with what is postulated in Barker's fetal programming hypothesis [7,24]. The increase in nutrients that reach the placenta causes changes in the baby's physiology and metabolism. These changes are programmed and increase the risk of overweight and obesity in childhood and adult life and are the beginning of multiple diseases [24].

In our study, maternal overweight and obesity on pregnancy it is associated with a higher level of plasma glucose in the third trimester of pregnancy, without reaching diagnostic levels for gestational diabetes. This alteration in glycemia causes epigenetic changes, which affect the development of the fetus causing metabolic alterations that accompany the baby throughout its life, contributing to the appearance of diseases in adult life, possibly being the greatest risk indicator for the development of overweight or obesity in the baby from birth to adulthood [25]. 

Concerning gynecological-obstetric history, there was a correlation between maternal overweight and obesity and a greater number of pregnancies and abortions, however, according to what was previously described, it is possible that this is due to the follow-up time of the study, since It seems to be more of an event of reverse causality [26], that is to say that in each pregnancy a weight gain is conditioned that the mother is unable to lose [27], in the same way this greater number of pregnancies can condition the number of abortions, although the latter can appear in response to higher glucose levels, insulin resistance and even a higher BMI [28].

An interesting fact is the association of maternal overweight or obesity with a lower number of prenatal consultations. This has several possible explanations, among those biological, is the difficulty in suspecting pregnancy in the initial stages on the part of the woman, since menstrual irregularities disorders are usually frequent in these patients due to the presence of polycystic ovary syndrome [29]. Furthermore, the increased abdominal diameter due to the adipose panicle makes it difficult for women to identify signs of uterine growth. Other possible conditions correspond more to the scope of care, since in Mexico, the conditions inherent to the training and practice of medicine, as well as the conditions of the health system, predisposes health personnel to be less empathetic and have borderline behaviors that sometimes may not adapt to a framework of good treatment of women who are overweight or obese, which implies that women reduce prenatal control appointments during their pregnancy consciously or unconsciously [30-32].

It is important to mention that maternal overweight or obesity prior to and during pregnancy has been shown to have important effects on the course and outcome of pregnancy, and on the short, medium and long-term development of the newborn. During pregnancy, exposure can cause a greater risk of suffering from various diseases, it can increase the probability of premature birth and in the newborn, it can cause a higher birth weight that can even reach macrosomia and that can continue until adult life [33-35]. Regarding breastfeeding, it has a protective effect against overweight and obesity on newborns throughout the baby’s life, however, it is unknown if the beneficial effect can reduce or counteract the alterations created by in utero exposure during pregnancy to maternal overweight and obesity [18,19,34].

It is known that breast milk has a wide capacity to modify its components always seeking to promote the baby's health, maternal characteristics may cause some of these changes [9,10]. Although maternal overweight and obesity may be factors that modify the bioactive components of breast milk [18,19], mammary gland has control mechanisms to reduce the infant's overexposure to components that could have a negative effect on its health, such as its apocrine and merocrine secretion system, which allows greater regulation of the compounds that reach breast milk as well as the large amount of bioactive substances with a beneficial effect in regulating the generation of adiposity and the control between hunger and satiety cycles [35].

However, whether the baby's development is affected by these modifications remains unclear, especially because long-term breastfeeding reduces the risk of overweight and obesity in those who receive it [19,33-36]. This research followed women with exclusive breastfeeding for 180 days, seeking to identify the possible effect of breast milk on the development of the baby, although a statistically significant difference was not identified between the weight (in grams) of the babies of mothers with and without overweight or obesity, if there was a tendency towards a higher weight in the babies of mothers who were overweight or obese, in addition to a greater variability in weight according to the 95%CI for the mean. In the same way the WHO indicators were used, where differences were found in the 60-, 120-, 150- and 180-day follow-ups in weight for length, being greater for the overweight and obese group.

Despite the above, it is possible that the cause of this weight increase in the baby is a combination between gestational exposure to maternal overweight or obesity [25] in counterpart to the beneficial biochemical modifications of breast milk derived from this same exposure [18,19,37-39]. Eventually, in the long term they will join the environment in which the child develops and that if not modified will perpetuate bad eating habits and a constant sedentary lifestyle, increased a risk of a higher BMI in childhood and adulthood [40].

Regardless of the mechanism by which the baby is affected, in the long term, maternal overweight or obesity on pregnancy has an influence on the baby's development [38], so preconception care is essential to identify and treat these metabolic alterations in time in the mother.

The limitations of the present study are related to the small sample size, due to the costs and difficulties of follow-up in a wide geographic region such as that used for the study, in addition to the short follow-up period, since the effect of maternal overweight and obesity should be evaluated for as long as possible and take into account the beneficial effect of breastfeeding, the characteristics and habits of the family where children grow and develop. An important point to study is the modifications in quantities of nutritional compounds (lipids, proteins and carbohydrates), as well as bioactive factors (hormones, interleukins, etc.) that are modified in obesity and that can be identified directly in breast milk.

Finally, the findings imply that in overweight and obese mothers the practice of breastfeeding should be promoted and protected, since receiving breast milk can counteract the negative effects of gestational exposure to metabolic alterations caused by a higher BMI. It will always be much more beneficial for both mother and baby to provide and receive breastfeeding in the short and long term [40]. Rather, the task is preconception care of maternal weight by the multidisciplinary health team to reduce the prevalence of women who are overweight and obese during pregnancy.

## Conclusions

Maternal overweight and obesity during pregnancy have a negative effect on the mother's health and the baby's weight gain in its weight-for-length classification during the first six months of life. 

Although breastfeeding has been shown to have a positive effect on the growth of the baby, exposure to a higher maternal body mass index during pregnancy triggers important metabolic alterations on babies that promote the development of diseases. Therefore, we must promote and protect breastfeeding, especially knowing that alterations in maternal weight are one of the causes that make it difficult to start and continue breastfeeding.

It is important to establish weight control guidelines for women who wish to become pregnant to reduce the negative effects on the mother and offspring caused by overweight and obesity.
